# Enhanced therapeutics of cabazitaxel via polycarboxylate conjugation: improved solubility, safety, and antitumor efficacy

**DOI:** 10.3389/fimmu.2025.1680710

**Published:** 2025-11-26

**Authors:** Lina Mao, Yan Zhang, Na Zhu, Xueming Wang, Tianjun Liu

**Affiliations:** Tianjin Key Laboratory of Biomedical Materials, Institute of Biomedical Engineering, Chinese Academy of Medical Sciences and Peking Union Medical College, Tianjin, China

**Keywords:** cabazitaxel, polycarboxylic acid modification, microtubule stabilization, thymic toxicity, tumor targeting

## Abstract

**Introduction:**

Cabazitaxel (CTX) is a potent anticancer agent whose clinical utility is severely limited by poor aqueous solubility and severe systemic toxicity. To overcome these challenges, we designed and synthesized two water-soluble polycarboxylate conjugates, CTX-DTPA and CTX-TTHA.

**Methods:**

The conjugates were characterized via NMR and mass spectrometry. Their hydrophilicity was assessed by lipid-water partition coefficient. Antitumor activity was evaluated in vitro across multiple cancer cell lines and in vivo using xenograft and immunocompetent models. Mechanisms were investigated via molecular docking, immunofluorescence, and tubulin polymerization assays. Safety profiles were assessed through hemocompatibility, myelosuppression, and thymic toxicity evaluations. Pharmacokinetics and biodistribution were analyzed in SD rats.

**Results:**

The derivatives exhibited potent antitumor activity comparable to unmodified CTX, effectively inducing apoptosis and G2/M cell cycle arrest. Mechanistic studies revealed moderately reduced binding affinity to β-tubulin but more sustained microtubule stabilization. Remarkably, the conjugates demonstrated a 64-fold improvement in hemocompatibility, significantly minimized myelosuppression, and preserved thymic architecture and immune function. Pharmacokinetic analysis showed prolonged circulation, efficient clearance, and drastically diminished off-target tissue accumulation for CTX-DTPA.

**Discussion:**

This study establishes polycarboxylate conjugation as a promising strategy for developing safer and more effective chemotherapeutic agents through rational molecular design, successfully decoupling antitumor efficacy from systemic toxicity.

## 1 Introduction

Cabazitaxel (CTX), a potent semi-synthetic microtubule inhibitor, is a key therapeutic option for treating advanced, taxane-resistant cancers (1–4). However, its formidable clinical utility is severely hampered by formulation challenges stemming from an inherent lack of aqueous solubility (5). The requisite solubilizing agents, polysorbate 80 and ethanol, are not pharmacologically inert; they are directly implicated in a spectrum of serious adverse effects, including acute hemolysis, hypersensitivity reactions, and cumulative organ toxicity, which collectively contribute to narrow therapeutic index of CTX (6).

In response, the scientific community has largely pursued a drug delivery system (DDS) paradigm (7–10). Strategies such as lipid emulsions (11, 12), polymeric nanoparticles (13, 14), albumin-bound complexes (15), and various other nanocarriers have been explored to physically encapsulate CTX (16). While preclinical successes have been reported, these carrier-based approaches often have intrinsic limitations: complex and poorly reproducible manufacturing, low drug-loading capacity, instability during storage leading to premature drug release, and potential immunogenicity of the carrier materials themselves (17–20). These challenges underscore that physical encapsulation may not be the most robust or clinically translatable path forward.

Herein, we propose a paradigm shift from physical encapsulation to rational chemical redesign. Instead of constructing a vehicle for the drug, we directly engineered the drug molecule itself. We report a novel strategy of polycarboxylic acid conjugation to create two inherently water-soluble CTX derivatives: CTX-DTPA and CTX-TTHA. This chemical modification fundamentally alters the hydrophilicity of CTX, transforming it into a new molecular entity that eliminates the need for toxic solvents or complex nanocarriers.

In addition to addressing the paramount issue of solubility, this study rigorously investigated whether this structural redesign can dissociate the antitumor efficacy of CTX from its systemic toxicity. We place particular emphasis on thymic toxicity—a critical yet underappreciated side effect of taxane therapy that can compromise long-term immune competence. Through comprehensive *in vitro* and *in vivo* evaluations, we demonstrate that this conjugation strategy not only preserves potent anticancer activity but also confers a dramatically enhanced safety profile, offering a promising blueprint for next-generation, immunocompatible chemotherapeutics.

Methods

### 1.1 Synthesis of CTX-TTHA or CTX-DTPA

The CTX-TTHA and CTX-DTPA conjugates were synthesized through polycarboxylic acid conjugation. First, triethylenetetraminehexaacetic acid (TTHA, 1.48 g) was activated with acetic anhydride (2 mL) and pyridine (1.89 g) at 45°C for 4 h. The intermediate was washed with acetic anhydride and ether, then dried under vacuum. For conjugation, the activated intermediate (0.45 g) was dissolved in DMF with Na_2_SO_4_•10H_2_O (0.035 g) at 70°C for 4 h, followed by addition of cabazitaxel (0.75 g), DMAP (0.012 g), and TEA (140 μL) with 24 h stirring. The products were precipitated with cold ether and purified by semi-preparative HPLC, yielding >97% pure conjugates as confirmed by ¹H NMR and mass spectrometry. Diethylenetriaminepentaacetic acid (DTPA) was treated following the identical procedure.

### 1.2 Lipid-aqueous partition coefficient

CTXs were dissolved in octanol, and an equal volume of water was added, followed by shaken at 100 rpm for 24 h at room temperature. The upper octanol phase and lower water phase were separated by centrifugation. The supernatant was analyzed by HPLC (Bruker SolanX 70 FT-MS; Agilent 6540TOF) (21).

### 1.3 Cytotoxicity

Human cancer cell lines were cultured in basic medium (10% fetal bovine serum (FBS)+1% double antibody solution) (Biological Industries) at 37°C in a 5% CO_2_ incubator. Effects of CTXs on cell proliferation and viability were measured by an MTS (Promega Corporation) assay. Absorbance values at 490 nm were measured using a microplate reader (Thermo Scientific, Varioskan Flash) (22).

### 1.4 Cell cycle arrest

Cells were incubated with drugs for 24 h. Fixed cells were incubated with RNase A and stained with propidium iodide (PI). DNA content was determined in a flow cytometer (BD FACS CaliburTM Flow Cytometer, USA) (23).

### 1.5 Molecular docking simulations

The molecular interactions between cabazitaxel (CTX) derivatives and human β-tubulin isotypes were investigated through molecular docking simulations using AutoDock 4.2 software (24). The crystal structure of human β-tubulin (PDB ID: 5SYF; resolution: 3.5 Å) was obtained from the RCSB Protein Data Bank. Structural preparation involved: (1) removal of water molecules and heteroatoms using PyMOL 2.5.2, and (2) addition of polar hydrogen atoms and assignment of Gasteiger charges using AutoDock Tools 1.5.7.

Prior to docking, all CTX derivatives were energy-minimized and converted to PDBQT format using Chem3D and AutoDock Tools, with rotatable bonds properly defined. Docking simulations were performed with AutoDock Vina 1.2.0 employing a semi-flexible approach (rigid protein, flexible ligands). A grid box of dimensions 39.8 × 40.5 × 42.0 Å³ was centered on the binding pocket coordinates (x = 327.007, y = 461.105, z = 364.004). Key docking parameters included: exhaustiveness = 32, number of modes = 10, and energy range = 3.0 kcal/mol.

The docking protocol was validated through redocking of the native ligand, yielding an acceptable root-mean-square deviation (RMSD) of 1.5 Å. The lowest-energy conformation from each docking simulation was selected for detailed interaction analysis, with molecular visualization and analysis performed using PyMOL.

### 1.6 Immunofluorescence

DU145 and PC-3 cells were fixed, and incubated with the rabbit monoclonal anti-α tubulin antibody (1:400, ab52866; Abcam) for 1 h, and thereafter with Alexa Fluor 488 conjugated goat anti-rabbit secondary antibody (1:500, ZF-0511; ZSGB-BIO) for 1 h. After DAPI staining, the fluorescently stained cells were visualized by confocal microscopy (Zeiss LSM710, Germany).

### 1.7 Purified tubulin polymerization

A tubulin polymerization assay kit (Cytoskeleton, BK006P) was used. Briefly, 10 μl of CTXs with a concentration of 100 μM in the general tubulin buffer and 100 μl of tubulin (3 mg/ml) in TP buffer were added on ice. The 96-well plate was immediately placed in a microplate reader at 37°C.

### 1.8 Hemolysis test

The hemolytic potential of CTX formulations was evaluated using rat red blood cells (RBCs). Briefly, whole blood was collected from the abdominal aorta of anesthetized SD rats into ACD anticoagulant (blood:ACD = 1:4, v/v). The blood was diluted with normal saline (4:5, v/v) and centrifuged at 450 g for 10 min. The RBC pellet was washed three times with normal saline until the supernatant was clear. The washed RBCs were then resuspended in normal saline to prepare a 2% (v/v) suspension.

For the assay, 500 μL of the 2% RBC suspension was mixed with an equal volume of serially diluted drugs or controls in 1.5 mL tubes. The controls included: Negative control (0% hemolysis): normal saline. Positive control (100% hemolysis): distilled water. Solvent control: the vehicle for unmodified CTX (2.5% Tween 80 + 2.5% anhydrous ethanol + 95% normal saline). The mixtures were incubated at 37 °C for 1.5 h and then centrifuged at 450 g for 5 min. Subsequently, 150 μL of the supernatant from each tube was transferred to a 96-well plate, and the absorbance at 545 nm (OD_545_) was measured using a microplate reader. The percentage of hemolysis was calculated using the following formula: Hemolysis (%) = [(OD_sample_ - OD_negative control_)/(OD_positive control_ - OD_negative control_)] × 100.

### 1.9 Tumor models and drug administration

All animal experiments were conducted in accordance with protocols approved by the Institutional Animal Care and Use Committee of Peking Union Medical College. Male BALB/c nude mice (6–8 weeks old) and female KM mice (6–8 weeks old) were purchased from Charles River Laboratories (Beijing, China) and maintained under specific pathogen-free conditions. Drug doses were optimized based on preliminary toxicity studies to achieve comparable therapeutic effects across different tumor models.

DU145 Prostate cancer model: DU145 cells (3×106 cells/mouse) were subcutaneously inoculated into the right flank of BALB/c nude mic. When tumors reached approximately 100 mm³, mice were randomly divided into four treatment groups (n=6/group): (a) Vehicle control; (b) CTX (3 μmol/kg); (c) CTX-TTHA (3 μmol/kg); (d) CTX-DTPA (3 μmol/kg). Treatments were administered via tail vein injection three times at 3-day intervals. Major organs were collected for histopathological examination after the final administration.

MCF-7 Breast Cancer Model: Female BALB/c nude mice bearing MCF-7 xenografts (2×10^6^ cells/mouse) were randomized into treatment groups as described above, with an adjusted dose of 9.6 μmol/kg for all CTX formulations.

H22 hepatoma model: Female KM mice were subcutaneously implanted with H22 cells (2×10^6^ cells/mouse) and assigned to six groups: (a) Vehicle control; (b) CTX (9.6 μmol/kg in 5% glucose with 2.5% ethanol/2.5% Tween 80); (c) CTX-TTHA (9.6 μmol/kg in saline); (d) CTX-DTPA (9.6 μmol/kg in saline); (e) CTX-TTHA (19.1 μmol/kg in saline); (f) CTX-DTPA (19.1 μmol/kg in saline).

Treatments were administered every other day for four doses (three doses for CTX group due to weight loss >15%). Peripheral blood samples were collected for complete blood count analysis using an automated hematology analyzer.

Drug doses were optimized based on preliminary toxicity studies and the principle of molar equivalence to achieve comparable therapeutic effects across different tumor models. The doses for CTX-DTPA and CTX-TTHA were calculated to deliver an equimolar amount of the cabazitaxel moiety relative to the unmodified CTX.

### 1.10 Thymic cell apoptosis analysis

Gently mix 1×10^6^ thymocytes with FITC Anexin V (5 μl) and PI (10 μl), and incubate in the dark for 15 minutes, RT. Analyze by flow cytometry. Thymocytes were stained with anti-mouse CD3e cyanine 5.5 (eBioscience, 85-45-0031-82), anti-CD4 PE (eBioscience, 85-12-0042-82), anti-CD8a FITC (4°C 30 min) and detected by flow cytometry.

### 1.11 Pharmacokinetic methods

Male and female SD rats (200–220 g) received intravenous injections of CTX or CTX-DTPA (6 μmol/kg). CTX was dissolved in 2.5% Tween 80/2.5% EtOH/saline, while CTX-DTPA used saline alone. Blood samples were collected at 5 min to 24 h post-dose. Tissues (heart, liver, spleen, lung, kidney, thymus) and excreta were collected at 15 min, 2 h, 4 h, and 12 h. Plasma and tissue samples were processed by liquid-liquid extraction with n-butanol and analyzed via LC-MS/MS (Agilent Poroshell 120 EC-C18 column; 0.05% formic acid/acetonitrile gradient). Pharmacokinetic parameters were calculated using DAS 2.0 via non-compartmental analysis.

### 1.12 Statistical analysis

All the data were expressed as mean ± SD, and a one-way analysis of variance (ANOVA) was performed. P<0.05 was considered to be statistically significant.

## 2 Results

### 2.1 Synthesis of the CTX-TTHA and CTX-DTPA conjugates

**Figure 1:**
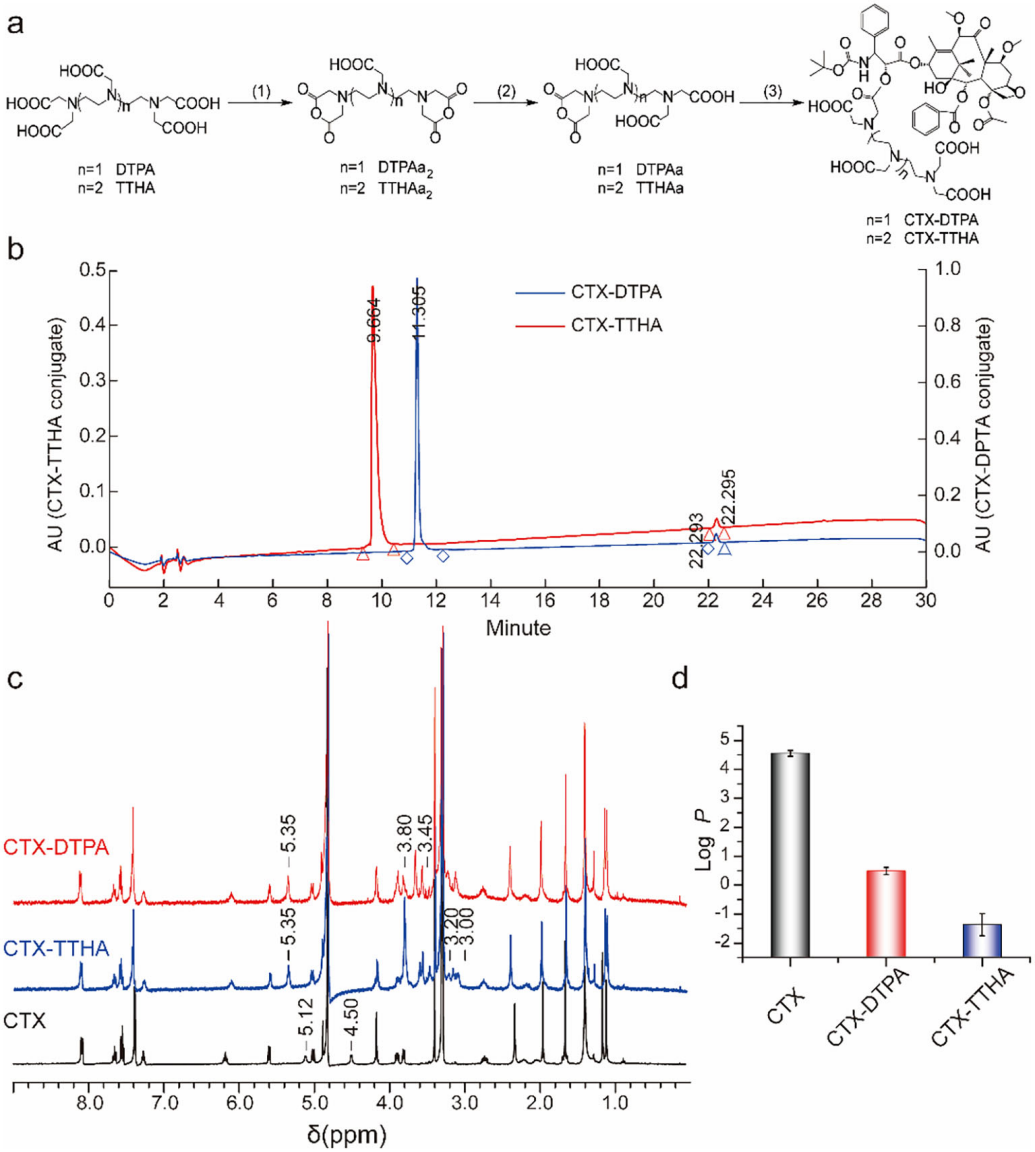
Compounds CTX-TTHA and CTX-DTPA. **(a)** Synthesis of water-soluble cabazitaxel compounds. Note for reagents and conditions. (1) Acetic anhydride, pyridine, 45°C, 4 h. (2) Dimethylformamide, 70°C, 4 h. (3) Cabazitaxel, 4-dimethylaminopyridine, triethyl amine, RT, 24 h. **(b)** HPLC trace of CTX-DTPA and CTX-TTHA conjugate. Running in a Waters C18 column (Kromasil, 5μm, 4.6 × 250 mm) with a flow of 1 mL/min of 0.05% methanoic acid in acetonitrile. The HPLC data was quantified by the integrated area under the peak at an absorbance of 227 nm. 1H NMR (400 MHz, Methanol-D4) **(c)** and lipid water distribution coefficients **(d)** of CTXs.

The water-soluble CTX derivatives, CTX-DTPA and CTX-TTHA, were successfully synthesized through polycarboxylic acid conjugation (**Figure 1a**). Structural confirmation of the synthesized compounds was achieved using ^1^H NMR spectroscopy (**Figure 1c**) and mass spectrometry (**Supplementary Figures S1**, **S2**). The ^1^H NMR spectra revealed a notable downfield shift of the -CH proton signal at the 2’-position of cabazitaxel, from 5.12 ppm (CTX) to 5.35 ppm (CTX-DTPA) or 5.35 ppm (CTX-TTHA). Concurrently, the characteristic hydroxyl proton peak at 4.50 ppm (2’-OH) in CTX disappeared in the derivatives, while new peaks emerged at 3.45–3.80 ppm and 3.00–3.20 ppm, corresponding to the -N-CH_2_-COOH- and -N-CH_2_-CH_2_-N- moieties of the DTPA and TTHA frameworks, respectively.

HPLC analysis confirmed the high purity of the conjugates (~97%, **Figure 1b**), with MALDI-TOF mass spectrometry yielding the following molecular ions: [CTX-DTPA-Na]^+^ at m/z 1233.4953, [CTX-DTPA-2Na]^+^ at m/z 1255.4793 (**Supplementary Figure S1**), [CTX-TTHA-Na]^+^ at m/z 1334.5429, and [CTX-TTHA-2Na]^+^ at m/z 1356.5824 (**Supplementary Figure S2**).

The lipid-water partition coefficient assay demonstrated a significant enhancement in hydrophilicity for the derivatives. The logP values decreased from 4.55 ± 0.10 (CTX) to 0.49 ± 0.12 (CTX-DTPA) and -1.37 ± 0.38 (CTX-TTHA) (**Figure 1d**), confirming the order of water solubility as CTX < CTX-DTPA < CTX-TTHA. These results demonstrate that polycarboxylic acid modification significantly improves the aqueous solubility of cabazitaxel while preserving its structural integrity.

### 2.2 *In vitro* anticancer efficacy of CTX derivatives

The cytotoxic effects of CTX-DTPA and CTX-TTHA were evaluated across a panel of human cancer cell lines (A549, Bel-7402, DU145, Fadu, H358, H460, H520, HCT116, HT-29, LN229, MCF-7, PC-3, SK-OV-3) and two normal cell lines (L-02, 3T3). Both derivatives exhibited dose-dependent cytotoxicity comparable to unmodified CTX, with no statistically significant differences in IC_50_ values (p > 0.05) (**Table 1**). These results confirm that polycarboxylate conjugation preserves the antitumor potency of cabazitaxel *in vitro*.

**Table 1:** IC_50_ of CTXs on cells (nM).

Cells		CTX	CTX-DTPA	CTX-TTHA
Tumor cell lines	A549	2.5 ± 1.2	3.0 ± 1.1	3.3 ± 1.6
	Bel-7402	2.8 ± 0.5	2.9 ± 0.9	2.4 ± 0.4
	DU145	6.1 ± 2.4	5.1 ± 1.4	4.1 ± 2.2
	Fadu	1.6 ± 0.4	1.9 ± 0.6	2.3 ± 0.9
	H358	1.2 ± 0.6	1.6 ± 0.7	1.6 ± 0.4
	H460	2.4 ± 1.3	2.4 ± 1.1	1.9 ± 0.7
	H520	1.5 ± 0.7	2.0 ± 1.3	2.1 ± 0.6
	HCT 116	2.2 ± 1.1	3.7 ± 1.0	2.1 ± 0.9
	HT-29	7.4 ± 3.4	6.7 ± 2.9	8.4 ± 3.8
	LN229	8.5 ± 2.3	5.8 ± 2.2	7.6 ± 3.1
	MCF-7	5.3 ± 1.9	6.8 ± 2.4	5.5 ± 2.3
	PC-3	5.2 ± 1.7	4.1 ± 0.7	7.4 ± 2.8
	SK-OV-3	4.4 ± 0.9	5.4 ± 1.3	3.2 ± 0.8
Non-tumor cell lines	L-02	7.8 ± 1.5	8.8 ± 3.6	6.8 ± 0.4
	3T3	8.7 ± 3.2	8.3 ± 2.7	8.0 ± 3.3

Data was presented as mean ± SD, n=3.

### 2.3 Cell cycle arrest induced by CTX derivatives

**Figure 2:**
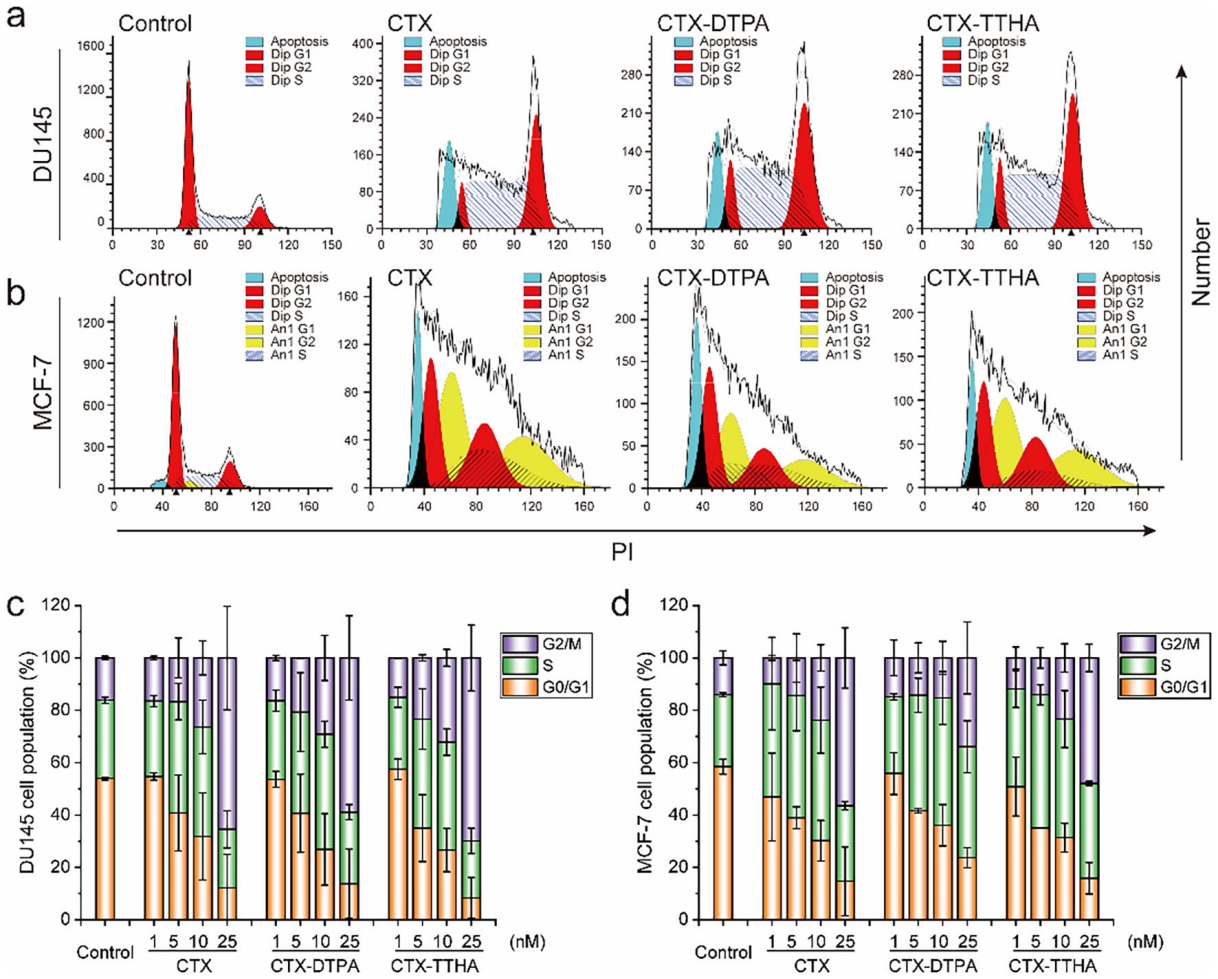
Cell cycle arrest by CTXs. Tumor cells were stained by PI and analyzed using flow cytometry. **(a, b)** Cell cycle results of DU145 cells **(a)** and MCF-7 cells **(b)** with 10 nM CTXs. The cell cycle distribution of tumor cells after CTXs treatment at different concentrations, **(c)** for DU145 cells, and **(d)** for MCF-7 cells. Data was presented as mean ± SD, n=3.

To investigate the impact of CTX-DTPA and CTX-TTHA on cell cycle progression, DU145 and MCF-7 cells were treated with varying concentrations (1–25 nM) of the derivatives and analyzed via flow cytometry. Both compounds induced dose-dependent G2/M phase arrest, mirroring the effects of unmodified CTX (**Figure 2**).

In DU145 cells, treatment with 1 nM CTX-DTPA or CTX-TTHA resulted in the arrest of 16.4 ± 1.0% and 15.1 ± 0.1% of the cells in G2/M, respectively, which was comparable to that observed with CTX (16.5 ± 0.7%). At the higher concentration of 25 nM, G2/M arrest increased significantly to 58.9 ± 16.1% (CTX-DTPA) and 69.9 ± 12.6% (CTX-TTHA), closely matching the effect of CTX (65.5 ± 19.9%) (**Figures 2a, c**).

Notably, in MCF-7 cells, the derivatives not only induced G2/M arrest but also promoted aneuploidy (**Figures 2b, d**, **Supplementary Figure S3**), suggesting additional disruption of mitotic fidelity. These results demonstrate that polycarboxylate conjugation preserves the ability of CTX to interfere with microtubule dynamics, leading to cell cycle arrest and aberrant chromosome segregation.

### 2.4 Molecular docking and microtubule interaction studies

**Figure 3:**
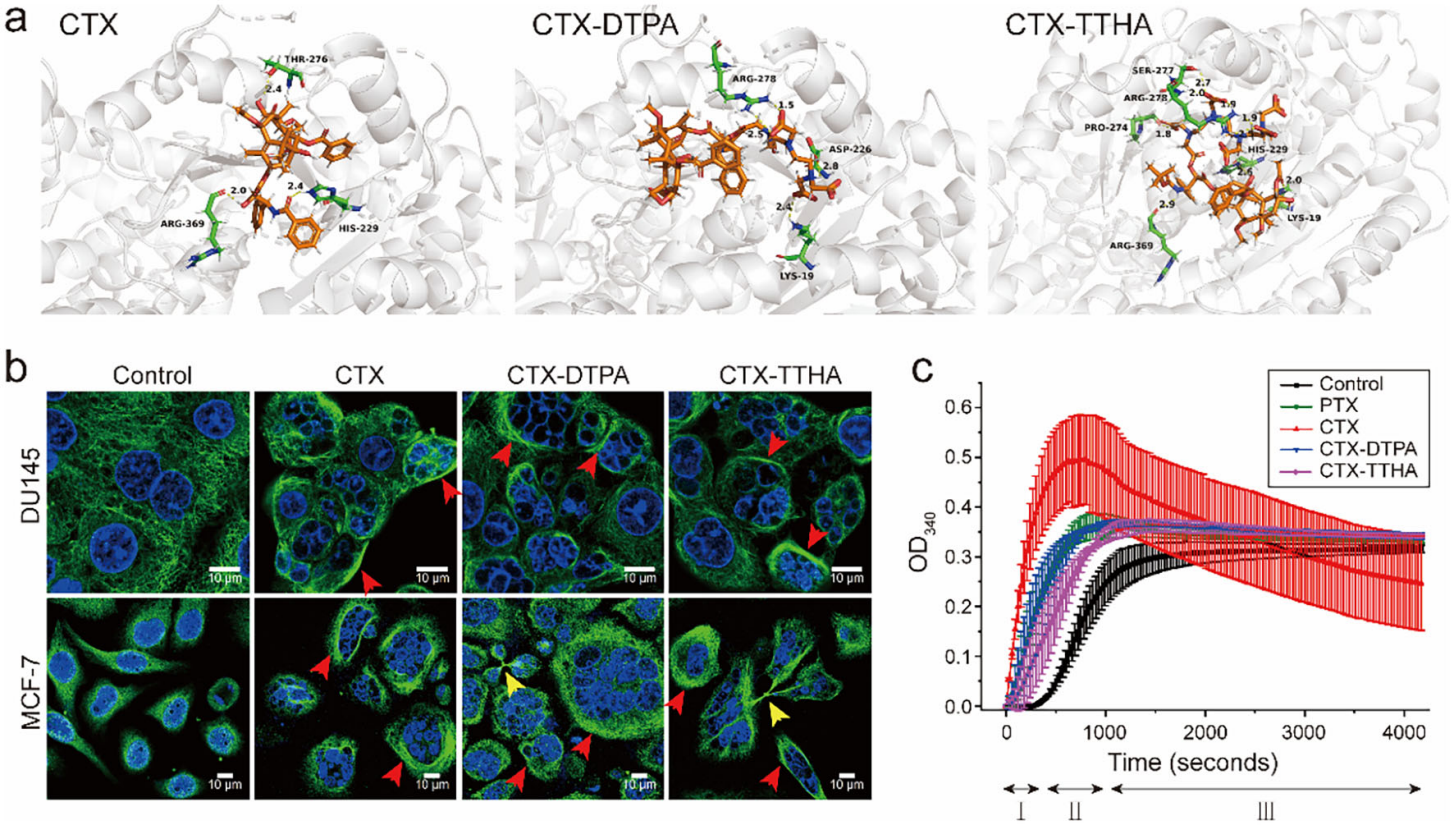
Effect on microtubules. **(a)** Molecular docking models of CTX derivatives with binding pocket of β-tubulin (PDBID: 5syf). Hydrogen bonds are represented by yellow dashed lines, with corresponding bond lengths labeled numerically. **(b)** Fluorescent micrographs of immunostaining for α-tubulin of DU145 and MCF-7 cells. Tumor cells treated with CTX, CTX-DTPA, and CTX-TTHA were stained with α-tubulin antibody (green), and DAPI (blue). The multinuclear structure was visualized, and polymerized microtubule bundles (red arrows) and multipolar splitting (yellow arrows) were indicated. Scale bar, 20 μm. **(c)** Tubulin polymerization reactions of CTXs. The control group illustrated the three phases of polymerization: I (nucleation), II (growth), III (steady-state). Data was presented as mean ± SD, n=3.

Molecular docking analysis revealed distinct binding profiles between native cabazitaxel (CTX) and its polycarboxylate-modified derivatives (CTX-DTPA and CTX-TTHA) with β-tubulin (**Figure 3a**). The unmodified CTX exhibited the strongest binding affinity (docking score: -8.37 kcal/mol), mediated through three critical interactions: (i) a 2.11 Å hydrogen bond between the C2’-hydroxyl group and Thr274, (ii) extensive hydrophobic contacts with His227, Pro272, and Arg276 residues, and (iii) stabilizing van der Waals interactions with Gly360 and Arg359. These molecular interactions collectively contribute to CTX’s potent microtubule-stabilizing activity.

Comparative analysis showed that the water-soluble derivatives maintained binding at the taxane site but with reduced affinities (CTX-DTPA: -5.80 kcal/mol; CTX-TTHA: -5.82 kcal/mol). Structural evaluation revealed that CTX-DTPA’s diethylenetriaminepentaacetic acid moiety caused complete loss of the Thr274 hydrogen bond due to steric constraints, while CTX-TTHA retained partial binding through a conserved His227 interaction (2.33 Å), albeit with compromised hydrophobic contacts. This calculated reduction in binding energy represents an intentional molecular trade-off to achieve superior pharmacokinetic properties.

Immunofluorescence studies revealed that 10 nM concentrations of both derivatives effectively disrupted microtubule networks in DU145 and MCF-7 cells (**Figure 3b**), inducing characteristic taxane effects including multinuclear formation and aberrant mitotic spindle organization. These cellular observations correlated well with tubulin polymerization assays, which showed that while all compounds enhanced nucleation phase kinetics, the derivatives exhibited more sustained stabilization patterns compared to the transient hyperpolymerization induced by unmodified CTX (**Figure 3c**). Notably, CTX-DTPA demonstrated polymerization kinetics (43.7 ± 8.0 mOD/min) comparable to paclitaxel, suggesting that polycarboxylate modification transforms CTX’s interaction with tubulin to produce more classical microtubule-stabilizing behavior.

These findings collectively demonstrate that while polycarboxylate conjugation modestly reduces tubulin binding affinity, it preserves the essential microtubule-targeting activity of CTX while conferring improved water solubility and altered polymerization dynamics. The derivatives’ ability to maintain antitumor efficacy despite reduced binding energy may be attributed to their enhanced cellular uptake and sustained drug release properties, as evidenced by the pharmacokinetic studies.

### 2.5 Ex vivo hemocompatibility assessment

**Figure 4:**
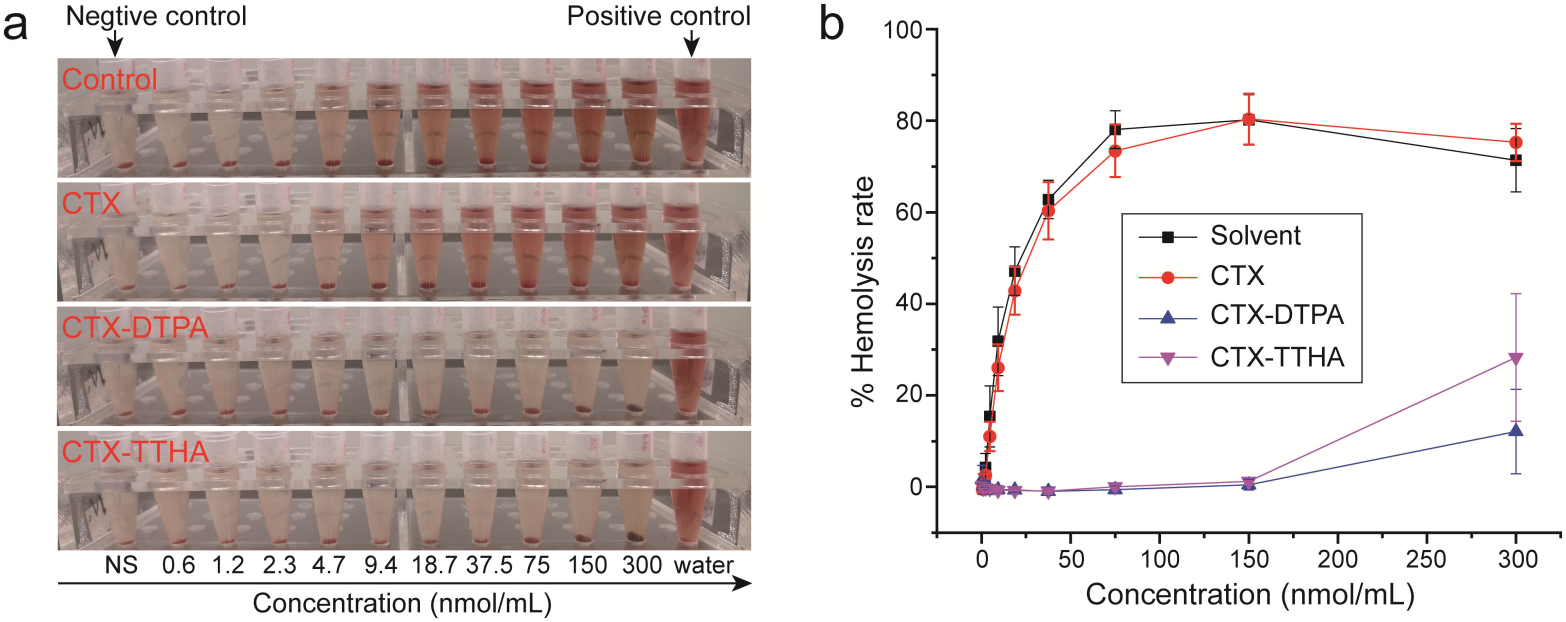
Hemolysis test of CTXs. **(a)** Hemolysis reactions of RBCs after 1.5 h incubation with different concentrations of CTXs. **(b)** The hemolysis rate of CTXs. Data was presented as mean ± SD, n=3.

The hemolytic potential of CTX derivatives was systematically evaluated using an ex vivo model of rat erythrocytes (**Figure 4**). Concentration-dependent analysis revealed striking differences in blood compatibility between the parent compound and its modified derivatives. Native CTX exhibited significant hemolytic activity, with hemolysis rates escalating from 11.0 ± 3.2% at 4.7 nmol/mL to 73.4 ± 5.8% at 75 nmol/mL.

In marked contrast, both CTX-DTPA and CTX-TTHA demonstrated substantially improved hemocompatibility profiles. The derivatives showed negligible hemolysis (<2%) at concentrations up to 150 nmol/mL - a 64-fold improvement over unmodified CTX. Even at the maximum tested concentration of 300 nmol/mL, hemolysis rates remained modest (12.1 ± 9.2% for CTX-DTPA and 28.3 ± 13.9% for CTX-TTHA), representing a dramatic reduction in erythrocyte membrane disruption compared to the parent compound.

### 2.6 Therapeutic efficacy and safety profile of CTX derivatives in immunodeficient mouse models

**Figure 5:**
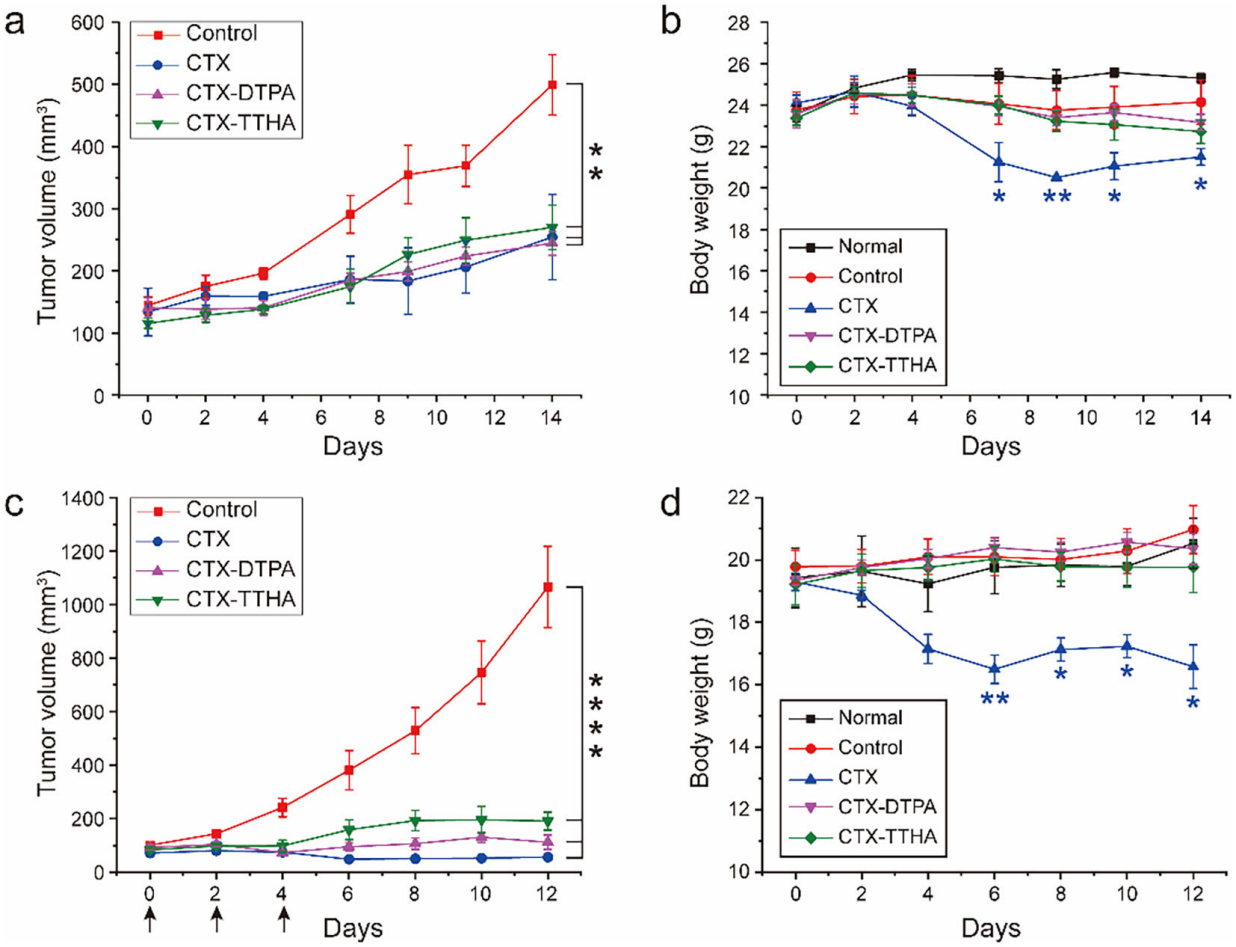
The impact of CTX on the DU145 and MCF-7 tumor models. **(a)** The volume growth curve of mice carrying DU145. **(b)** Body weight changes in different groups of mice carrying DU145. (Mean ± SD, n=6, compared to the normal group, *p<0.05, **p<0.01). **(c)** The volume growth curve of mice carrying MCF-7. The black arrow indicates the point of administration. **(d)** Body weight changes in different groups of mice carrying MCF-7. Data was presented as mean ± SD, n=6. (*p<0.05, **p<0.01, ****p<0.0001 compared to the control group).

We conducted comprehensive evaluations of CTX-DTPA and CTX-TTHA using two well-established xenograft models. In the DU145 prostate cancer model, all treatment groups exhibited significant tumor growth inhibition (p<0.01 versus vehicle control), with final tumor volumes of 244 ± 32 mm³ (CTX-DTPA), 270 ± 28 mm³ (CTX-TTHA), and 254 ± 35 mm³ (CTX), compared to 500 ± 45 mm³ in controls (**Figure 5a**). This comparable efficacy profile confirms that structural modification preserves the antitumor activity of the parent compound.

The safety advantages of the derivatives became particularly evident in longitudinal monitoring. While CTX treatment caused >15% body weight loss by day 7, both modified compounds maintained animal weights within normal physiological ranges (**Figure 5b**).

In the MCF-7 breast cancer model, we observed tumor volume reductions to 111 ± 18 mm³ (CTX-DTPA) and 190 ± 22 mm³ (CTX-TTHA) versus 56 ± 12 mm³ for CTX at study endpoint (**Figure 5c**). The complete survival (100%) in derivative-treated groups compared to 83.3% survival with CTX (1/6 mortality) further highlights the enhanced safety profile. Notably, CTX-DTPA showed particularly promising results, achieving near-equivalent efficacy to CTX while completely avoiding the cardiac hypertrophy observed with the parent compound (**Supplementary Figure S4**). Throughout the treatment period, both CTX-DTPA and CTX-TTHA maintained stable body weights in MCF-7 tumor-bearing mice (**Figure 5d**), further confirming their improved safety profile over the unmodified CTX.

### 2.7 Therapeutic efficacy and safety evaluation in immunocompetent H22 tumor-bearing mice

**Figure 6:**
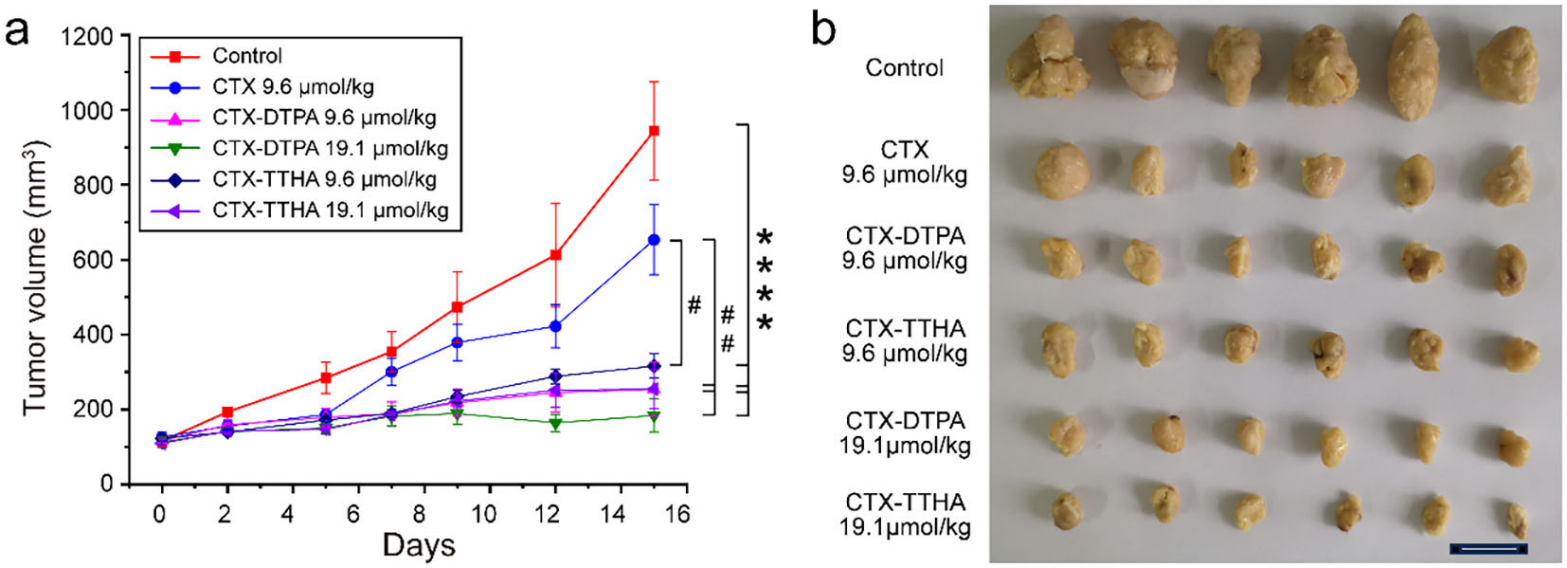
The effect of CTXs on H22 tumor model. **(a)** H22 tumor volume changes. Data was presented as mean ± SD, n=6 ****p<0.0001 *vs*. normal group, #p<0.05 *vs*. CTX group, ##p<0.01 *vs*. CTX group. **(b)** Macroscopic vies of implanted H22 tumors after treatment.

The antitumor efficacy of CTX derivatives was systematically evaluated in immunocompetent KM mice bearing H22 hepatocellular carcinoma tumors. Treatment with CTX-DTPA and CTX-TTHA at both standard (9.6 μmol/kg) and high (19.1 μmol/kg) doses demonstrated significant dose-dependent tumor growth inhibition compared to vehicle controls. Particularly noteworthy was the superior efficacy of high-dose CTX-DTPA, which achieved 80.5% tumor growth inhibition compared to the 30.8% inhibition observed with conventional CTX treatment. Macroscopic examination of excised tumors at study termination confirmed these quantitative findings (**Figure 6**).

### 2.8 Systemic toxicity evaluation in H22 tumor-bearing mice

**Figure 7:**
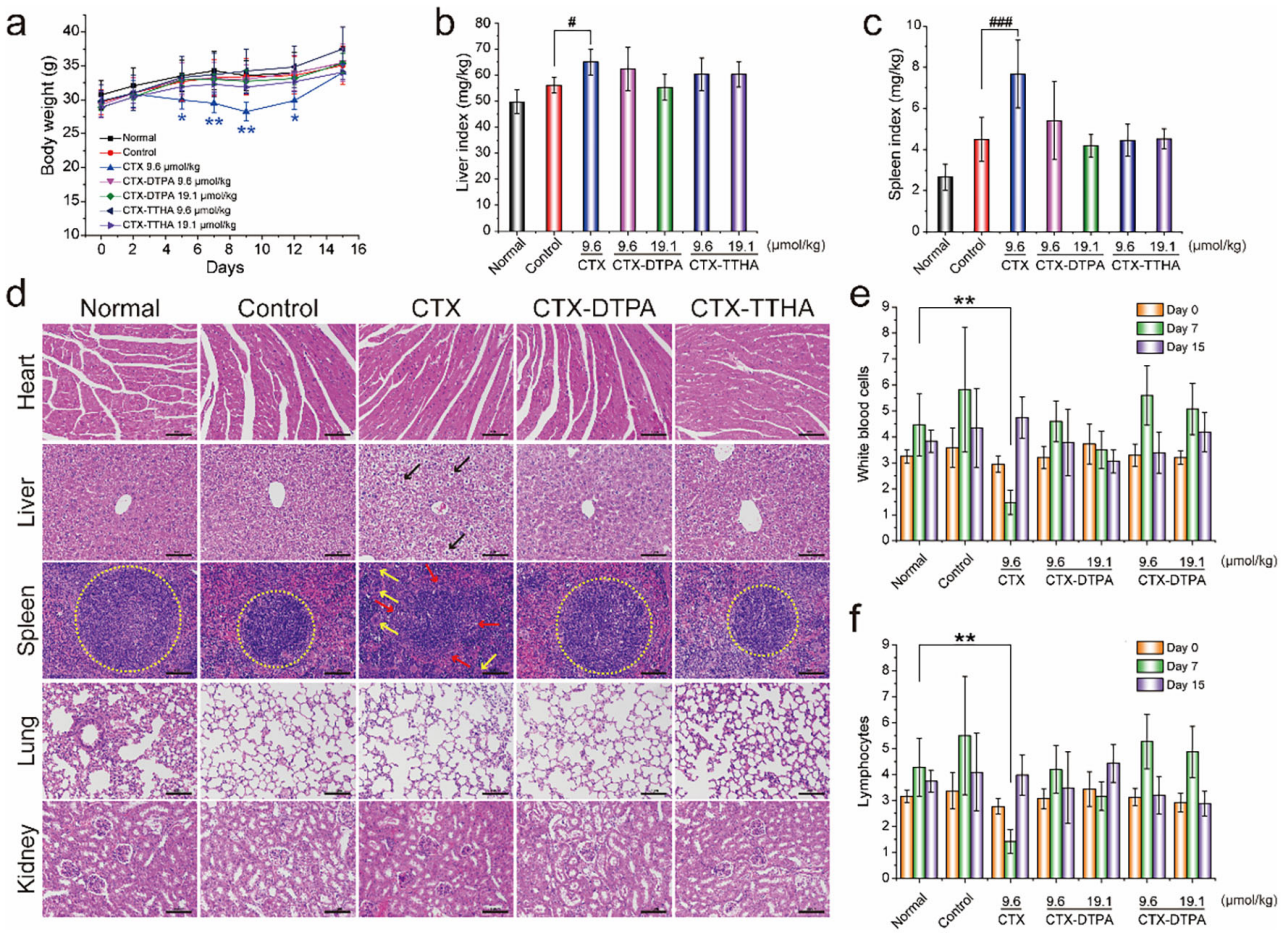
The effect of CTXs on H22 tumor-bearing mice. **(a)** Bodyweight changes. Liver **(b)** and spleen **(c)** indexes of mice in different groups (mean ± SD, n=6). #p<0.05, ###p<0.001 *vs*. control group. **(d)** H&E staining of main organs. Blank arrows indicated the balloon-like hepatocytes with swollen volume and vacuolar cytoplasm. The boundary of the red and white pulp of spleens (yellow circles). Yellow arrows indicated the increased macrophages. Scale bar, 100 μm. WBC **(e)** and lymphocyte **(f)** cell counts on day 7 and day 15. Data was presented as mean ± SD, n=6, *p < 0.05 *vs*. normal group, **p<0.01 *vs*. normal group.

The *in vivo* toxicity profiles of CTX-DTPA and CTX-TTHA were comprehensively assessed in immunocompetent H22 tumor-bearing mice. Animals treated with unmodified CTX exhibited significant (p<0.05) body weight loss (11-16%) and hepatosplenic toxicity, as evidenced by increased organ indices and histopathological abnormalities including ballooning hepatocyte degeneration and disruption of splenic architecture (**Figures 7a-d**). In striking contrast, both polycarboxylate-modified derivatives demonstrated markedly improved safety profiles at equivalent therapeutic doses.

Hematological analysis revealed that CTX caused severe myelosuppression, reducing white blood cell counts to 1.55 ± 0.17×10^9^/L (*vs* 4.46 ± 1.19×10^9^/L in controls, p<0.01) on day 7 post-treatment. Importantly, CTX-DTPA and CTX-TTHA maintained near-physiological hematological parameters throughout the treatment period, with WBC counts ranging from 3.50-5.60×10^9^/L across all dose groups (**Figures 7e, f**). This preservation of bone marrow function represents a critical therapeutic advantage over conventional CTX therapy.

### 2.9 Thymic preservation and immunocompatibility

**Figure 8:**
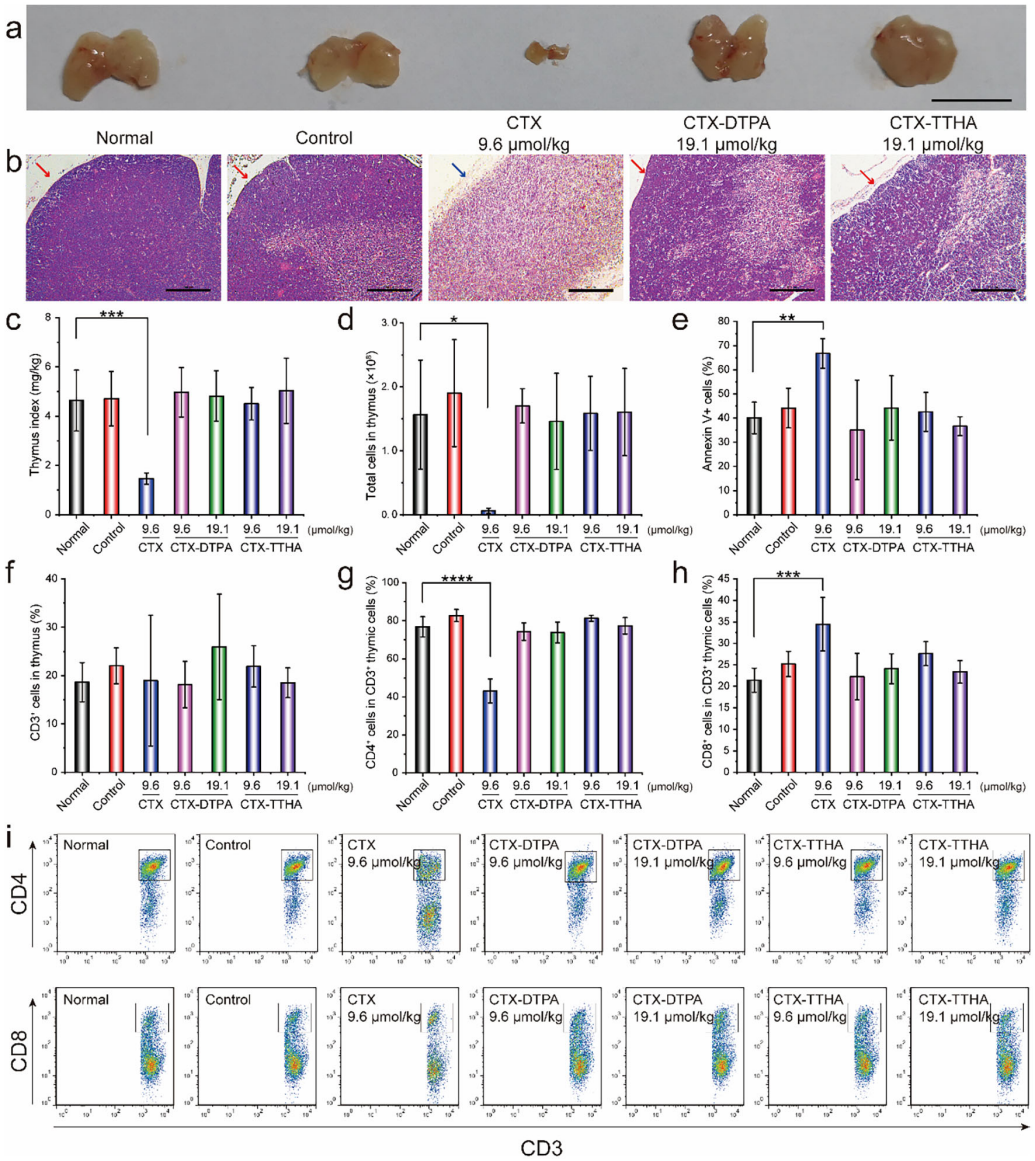
The effect of CTXs on the thymus in mice bearing H22 tumors. **(a)** Images of the thymus after 2 weeks of treatment. Scale bar, 1 cm. **(b)** H&E staining analysis of the thymus. The red arrow indicated the cortex of the thymus, and the blue arrow indicated the missing cortex. Scale bar, 100 μm. **(c)** Thymus index (mg/g). **(d)** Cell numbers of the thymus in different groups. **(e)** The apoptotic cells of the thymus were analyzed by flow cytometry. **(f)** The percentage of CD3^+^ thymic cells. The proportion of CD4^+^**(g)** and CD8^+^ cells **(h)** in CD3^+^ thymic cells. **(i)** The distribution of CD4^+^ and CD8^+^ cells in CD3^+^ thymic cells. Data was presented as mean ± SD. n=6. *p<0.05, **p<0.01, ***p<0.001, ****p<0.0001 *vs*. normal group.

The thymoprotective properties of CTX-DTPA and CTX-TTHA emerged as particularly noteworthy findings. While CTX administration resulted in profound thymic atrophy (31% of normal weight, p<0.05) and cortical depletion, both derivatives maintained normal thymic architecture with intact cortical-medullary differentiation (**Figures 8a–c**). Flow cytometric analysis confirmed the preservation of thymocyte populations (**Figures 8d, f**), with apoptosis rates in derivative-treated groups (35.1-44.2%) remaining comparable to controls and significantly lower than CTX-treated animals (66.8%, p<0.05) (**Figure 8e**). Furthermore, the modified compounds prevented the CTX-induced disruption of CD4^+^/CD8^+^ T cell ratios, maintaining the physiological balance critical for immune competence (**Figures 8g–i**).

These collective findings demonstrate that polycarboxylate modification successfully dissociates the antitumor efficacy of cabazitaxel from its dose-limiting toxicities, particularly the hematological and immunological adverse effects that have constrained clinical utility. The preservation of thymic structure and function suggests these derivatives may offer unique advantages for combination with emerging immunotherapies, potentially addressing a significant unmet need in contemporary cancer treatment paradigms.

To assess whether the preserved thymic architecture and cellularity translated to systemic immune homeostasis, we analyzed T-cell populations in the peripheral blood. Flow cytometric analysis revealed that the ratios of CD4^+^ and CD8^+^ T cells in all treatment groups, including those administered unmodified CTX, were comparable to those in normal, healthy mice (**Supplementary Figure S5**). This finding indicates that, at the time of measurement, the peripheral T-cell pool remained stable across all groups despite the severe thymic damage induced by CTX.

### 2.10 Pharmacokinetic and biodistribution profiles of CTX and CTX-DTPA

**Figure 9:**
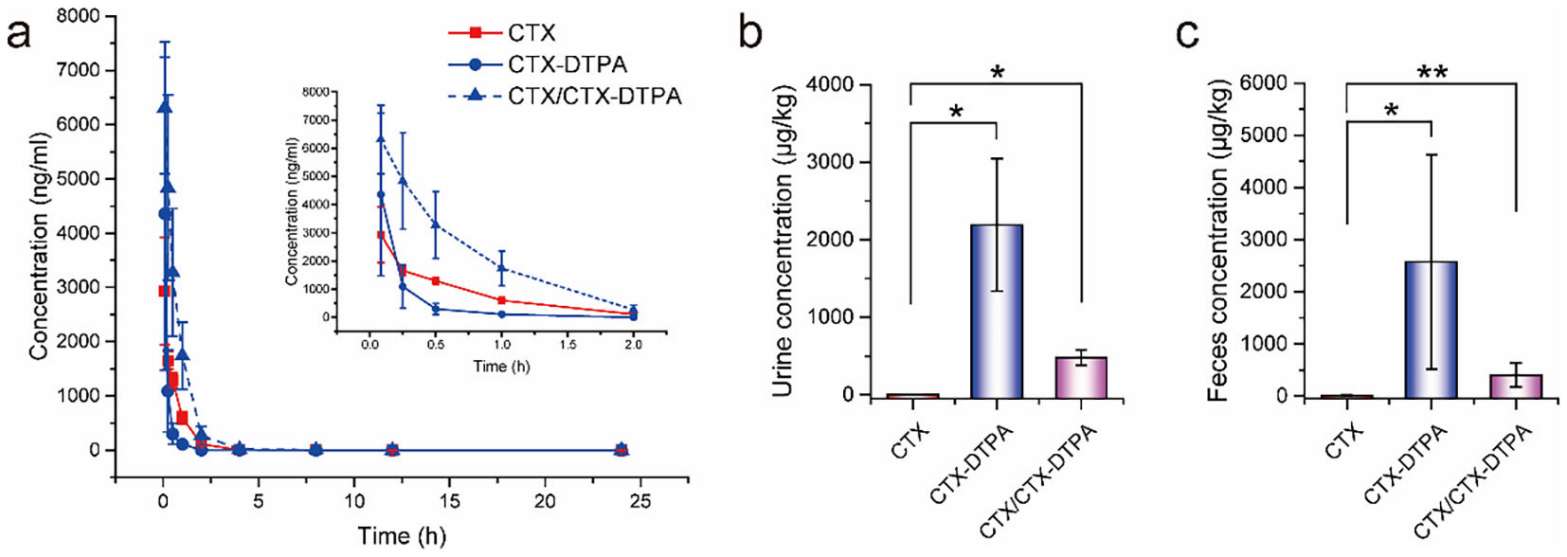
Pharmacokinetic and excretion profiles of CTX-DTPA versus CTX in SD rats. **(a)** Plasma concentration-time curves showing prolonged circulation of CTX-DTPA and its metabolite CTX/CTX-DTPA (6 μmol/kg IV dose). **(b)** Cumulative urinary excretion. **(c)** Cumulative fecal excretion. Data demonstrate enhanced elimination of CTX-DTPA (80% total excretion in 24 h) compared to CTX (<1%). Data was presented as mean ± SD (n=6). *p < 0.05 vs. CTX group, **p < 0.01 vs. CTX group.

The pharmacokinetic analysis revealed distinct profiles between CTX and its water-soluble derivative CTX-DTPA following intravenous administration (6 μmol/kg) in SD rats. CTX-DTPA demonstrated significantly prolonged systemic exposure compared to the parent compound, exhibiting a 5.1-fold longer elimination half-life (6.44 ± 0.00 h *vs*. 1.27 ± 0.09 h; p < 0.0001) and higher plasma exposure of the active metabolite CTX (CTX/CTX-DTPA) (AUC: 5157.71 ± 1650.87 *vs*. 1679.81 ± 193.83 μg/L•h; p < 0.0001) (**Table 2**, **Figure 9a**). The derivative showed rapid conversion to active CTX, achieving a 2.2-fold greater Cmax (6311.67 ± 1217.79 *vs*. 2931.67 ± 989.53 μg/L), suggesting sustained drug release characteristics.

**Table 2:** Non-compartmental model parameters.

Parameters	Units	CTX group	CTX-DTPA group	
		CTX	CTX-DTPA	CTX/CTX-DTPA
C_max_	μg/L	2931.67 ± 989.53	4356.67 ± 2881.79	* 6311.67 ± 1217.79
t_1/2_	H	1.27 ± 0.09	**** 6.44 ± 0.00	**** 2.77 ± 0.00
AUC_0-t_	μg/L*h	1679.81 ± 193.83	1334.61 ± 776.86	**** 5157.71 ± 1650.87
MRT_0-t_	H	0.67 ± 0.09	1.04 ± 0.57	0.72 ± 0.02
CL	L/h/kg	3.01 ± 0.34	* 7.53 ± 4.66	1.51 ± 0.41
V_d_	L/kg	5.53 ± 0.81	*** 69.93 ± 43.28	* 6.04 ± 1.63

C_max_, maximum concentration; t_1/2_, half time; AUC, area under concentration-time curve; MRT, mean residence time, MMRT; CL, Clearance; V_d_, apparent volume of distribution.

CTX: Drug concentration of cabazitaxel in the CTX treatment group.

CTX-DTPA: Concentration of the parent drug CTX-DTPA in the CTX-DTPA treatment group.

CTX/CTX-DTPA: CTX metabolite derived from CTX-DTPA biotransformation.

*p<0.05, ***p<0.001, ****p<0.0001 *vs*. CTX.

Data was presented as mean ± SD, n=6.

Both CTX-DTPA and its metabolite displayed markedly reduced tissue accumulation, with organ concentrations 10- to 100-fold lower than CTX across all examined tissues (**Table 3**). Particularly notable differences were observed in the thymus (0.62 ± 0.05 *vs*. 87.84 ± 23.46 μg/kg at 15 min; p < 0.01) and spleen (0.39 ± 0.04 *vs*. 249.73 ± 38.31 μg/kg at 15 min; p < 0.001), demonstrating substantially diminished off-target tissue distribution.

**Table 3:** Tissue distribution concentration (μg/kg).

Tissue	Time	CTX group	CTX-DTPA group	
		CTX	CTX-DTPA	CTX/CTX-DTPA
Thymus	15 min	87.84 ± 23.46	** 0.62 ± 0.05	** 1.49 ± 0.02
	2 h	129.15 ± 50.01	* 0.10 ± 0.07	* 4.37 ± 5.02
	4 h	109.86 ± 41.75	* 0.05 ± 0.01	* 0.79 ± 0.28
	12 h	67.21 ± 4.15	**** 0.06 ± 0.04	**** 0.80 ± 0.42
Heart	15 min	43.23 ± 12.9	* 0.27 ± 0.04	* 2.38 ± 0.46
	2 h	24.56 ± 2.33	*** 0.07 ± 0.03	*** 2.27 ± 0.70
	4 h	15.97 ± 1.91	*** 0.04 ± 0.00	*** 1.26 ± 0.20
	12 h	12.59 ± 2.69	** 0.06 ± 0.03	** 0.41 ± 0.29
Liver	15 min	149.33 ± 7.27	*** 37.74 ± 16.89	*** 17.88 ± 1.12
	2 h	78.73 ± 10.63	** 7.21 ± 1.30	** 10.31 ± 7.28
	4 h	48.74 ± 11.46	* 6.74 ± 5.04	** 4.62 ± 3.48
	12 h	29.33 ± 11.48	* 0.65 ± 0.42	* 1.91 ± 1.12
Spleen	15 min	249.73 ± 38.31	*** 0.39 ± 0.04	** 5.49 ± 0.53
	2 h	159.81 ± 2.24	**** 0.09 ± 0.03	**** 8.23 ± 4.20
	4 h	112.94 ± 21.04	** 0.05 ± 0.02	** 2.61 ± 0.42
	12 h	54.03 ± 8.47	** 0.05 ± 0.03	** 1.43 ± 0.96
Lung	15 min	132.60 ± 15.04	*** 2.36 ± 1.29	*** 12.95 ± 5.49
	2 h	116.51 ± 4.61	**** 0.48 ± 0.50	**** 9.66 ± 5.84
	4 h	106.75 ± 0.42	**** 0.13 ± 0.01	**** 2.44 ± 0.01
	12 h	79.02 ± 30.87	* 1.10 ± 0.45	* 1.78 ± 0.82
Kidney	15 min	549.67 ± 12.02	**** 41.08 ± 9.73	**** 53.38 ± 7.21
	2 h	313.23 ± 47.52	** 4.96 ± 1.67	** 63.87 ± 43.92
	4 h	307.73 ± 41.53	*** 1.55 ± 0.31	*** 8.74 ± 0.10
	12 h	218.19 ± 15.93	**** 1.36 ± 0.84	**** 3.14 ± 1.66

CTX: Drug concentration of cabazitaxel in the CTX treatment group.

CTX-DTPA: Concentration of the parent drug CTX-DTPA in the CTX-DTPA treatment group.

CTX/CTX-DTPA: CTX metabolite derived from CTX-DTPA biotransformation.

*p<0.05, **p<0.01, ***p<0.001, ****p<0.0001 *vs*. CTX.

Data was presented as mean ± SD, n=6.

The markedly reduced tissue accumulation of CTX-DTPA and its metabolite, particularly in immune organs like the thymus and spleen (**Table 3**), can be primarily attributed to its enhanced systemic clearance and high hydrophilicity, which limits passive diffusion into cells.

### 2.11 Excretion and safety profile

The excretion profiles differed dramatically between the two compounds. While CTX showed minimal elimination (<1% of dose in 24 h), CTX-DTPA exhibited rapid clearance with approximately 80% of the administered dose excreted within 24 hours, primarily through renal (39.8 ± 12.7%) and fecal (43.5 ± 32.8%) routes. The derivative’s enhanced water solubility facilitated this efficient elimination, with urinary excretion dominated by intact CTX-DTPA (2191.14 ± 853.66 μg/kg) (**Figure 9b**) and fecal excretion containing both parent drug and metabolite (**Figure 9c**).

These results demonstrate that DTPA modification transforms the pharmacokinetic behavior of CTX, creating a compound with prolonged circulation, sustained release of the active drug, reduced tissue retention, and enhanced elimination clearance, which may translate to an improved therapeutic index and reduced systemic toxicity compared with those of the parent compound.

## 3 Discussion

The strategic redesign of cabazitaxel through polycarboxylic acid conjugation, resulting in CTX-DTPA and CTX-TTHA, represents a transformative approach that successfully decouples the potent antitumor efficacy of CTX from its dose-limiting systemic toxicities (25–31). Our findings comprehensively demonstrate that this chemical modification strategy not only conquers the fundamental challenge of aqueous solubility but also fundamentally reshapes the pharmacokinetic and safety profile of the drug, offering a promising path toward a safer and more immunocompatible chemotherapy (32, 33).

A pivotal and intriguing finding of our study is the resolved paradox between reduced target affinity and retained cytotoxicity. Molecular docking revealed that the polycarboxylate conjugation modestly reduced the binding affinity of the derivatives to β-tubulin. This apparent discrepancy, however, is convincingly explained by a multi-faceted compensatory mechanism. The dramatically improved hydrophilicity undoubtedly enhances drug availability and cellular uptake efficiency. More importantly, the tubulin polymerization assays indicated that the derivatives induce a distinct, more sustained microtubule stabilization pattern, resembling paclitaxel rather than the transient hyperpolymerization characteristic of native CTX (34). This suggests that the chemical modification has reprogrammed the drug’s pharmacodynamic profile, leading to altered yet highly effective target engagement. Coupled with the efficient bioconversion to active metabolites observed in pharmacokinetic studies, these factors collectively ensure uncompromised antitumor potency.

The cornerstone of the clinical potential for CTX-DTPA and CTX-TTHA lies in their exceptionally enhanced safety profile (35, 36). The 64-fold improvement in hemocompatibility directly addresses the acute infusion-related toxicity of the clinical formulation. Furthermore, the mitigation of severe myelosuppression highlights a critical advantage over the parent compound. Perhaps the most significant advance is the pronounced thymoprotective effect. We observed that while unmodified CTX caused profound thymic atrophy and disrupted intrathymic T-cell development, our derivatives preserved the architectural and cellular integrity of this primary immune organ. This finding is of paramount importance, as the thymus serves as the factory for T-cell neogenesis (37). Its preservation is a fundamental prerequisite for long-term immune competence and resilience, a feature particularly crucial in the era of combination therapies with immunotherapies (37). The fact that our derivatives maintain a normal peripheral T-cell profile further supports that the central protection of the thymus successfully translates to systemic immune homeostasis.

The remarkably reduced tissue accumulation, especially in the thymus and spleen, provides the mechanistic underpinning for the observed immunocompatibility and reduced organ toxicity. As elucidated by our pharmacokinetic data, this phenomenon is primarily driven by the synergistic effect of enhanced systemic clearance and reduced passive cellular uptake due to the high hydrophilicity of the conjugates. This altered biodistribution is a direct result of our chemical design strategy, which strategically avoids the extensive tissue retention of the highly lipophilic parent drug.

From a pharmaceutical development perspective, our direct chemical conjugation strategy offers distinct advantages over conventional nanocarrier-based delivery systems. It bypasses challenges such as drug leakage, low loading capacity, carrier-related immunogenicity, and manufacturing inconsistencies. The synthesis is reproducible, yields a well-defined molecular entity, and results in a formulation that is stable in aqueous solution. The successful dissociation of efficacy from toxicity achieved here underscores the power of rational molecular design over complex physical encapsulation for optimizing cytotoxic agents.

In conclusion, this study establishes polycarboxylic acid conjugation as a robust and transformative strategy for revitalizing cabazitaxel. The derivatives CTX-DTPA and CTX-TTHA embody a unique combination of water solubility, potent and sustained antitumor activity, and a markedly improved safety profile encompassing hematological and immunological protection. The mechanistic insights into their altered tubulin interaction and favorable pharmacokinetics challenge conventional structure-activity relationship paradigms. Future work will focus on further structural refinements and evaluating the potential of these immunocompatible derivatives in combination with modern immunotherapies. This approach paves the way for a new generation of chemotherapeutic agents designed for both uncompromising efficacy and system-wide safety.

## Data Availability

The original contributions presented in the study are included in the article/**Supplementary Material**. Further inquiries can be directed to the corresponding author.
