# Supplementary material for: Enhanced therapeutics of cabazitaxel via polycarboxylate conjugation: improved solubility, safety, and antitumor efficacy
Source: Front Immunol. 2025 Nov 26;16:1680710. doi: 10.3389/fimmu.2025.1680710 (PMC12689286; doi:10.3389/fimmu.2025.1680710)
Supplement: Supplementary file 1 [file Table1.doc]

Enhanced Therapeutics of Cabazitaxel via Polycarboxylate Conjugation: Improved Solubility, Safety, and Antitumor Efficacy

Lina Mao, Yan Zhang, Na Zhu, Xueming Wang, Tianjun Liu*

Tianjin Key Laboratory of Biomedical Materials, Institute of Biomedical Engineering, Chinese Academy of Medical Sciences & Peking Union Medical College, Tianjin, 300192, China

*Corresponding author: liutj@bme.cams.cn

**Supporting Information**


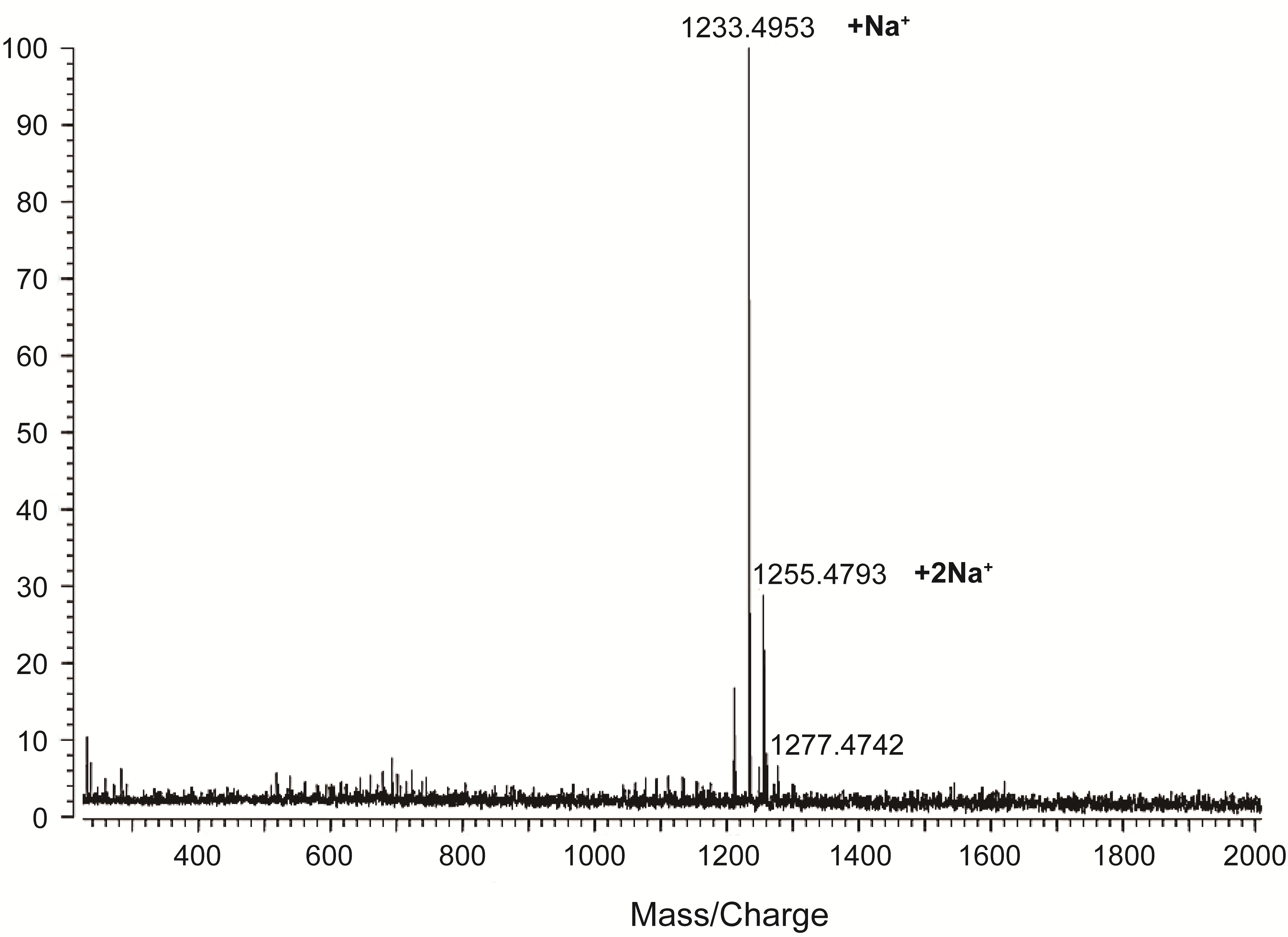


**Figure S1.** HR-MS of CTX-DTPA.


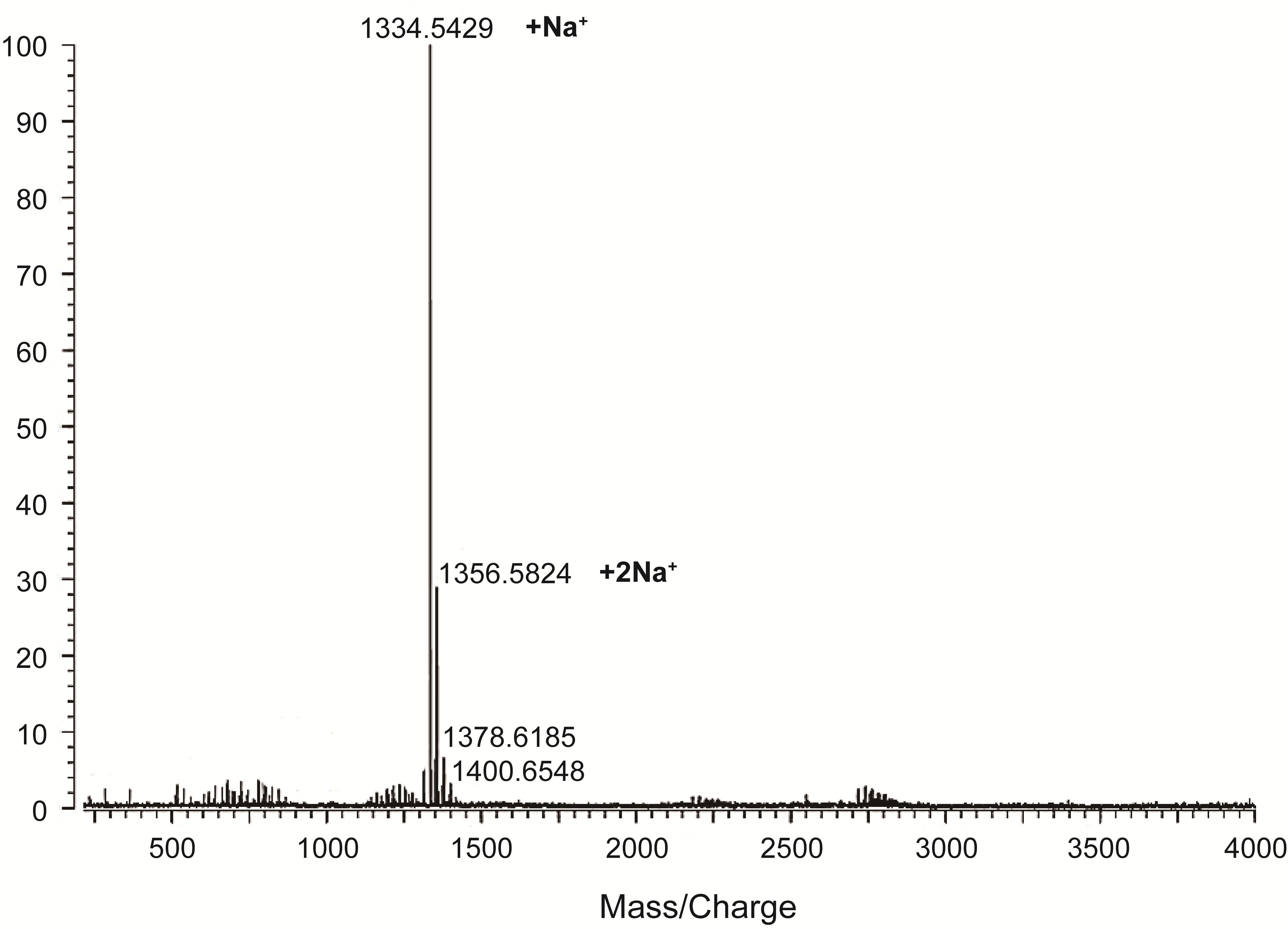


**Figure S2.** HR-MS of CTX-DTPA.


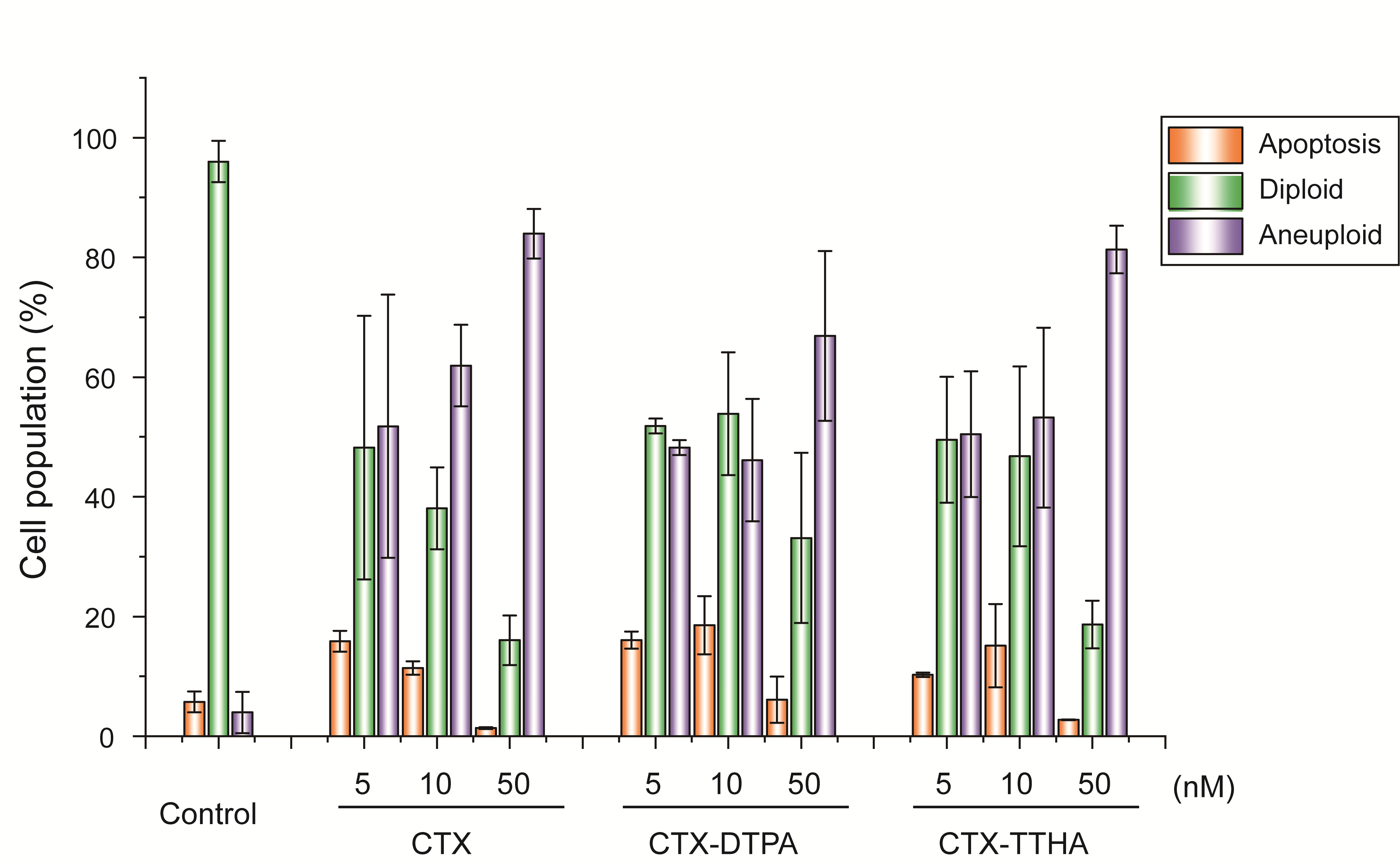


**Figure S3.** Cell cycle analysis of MCF-7 (mean±standard deviation, n=3). Data were presented as mean ± SD (n=6). *p＜0.05 vs. control group.


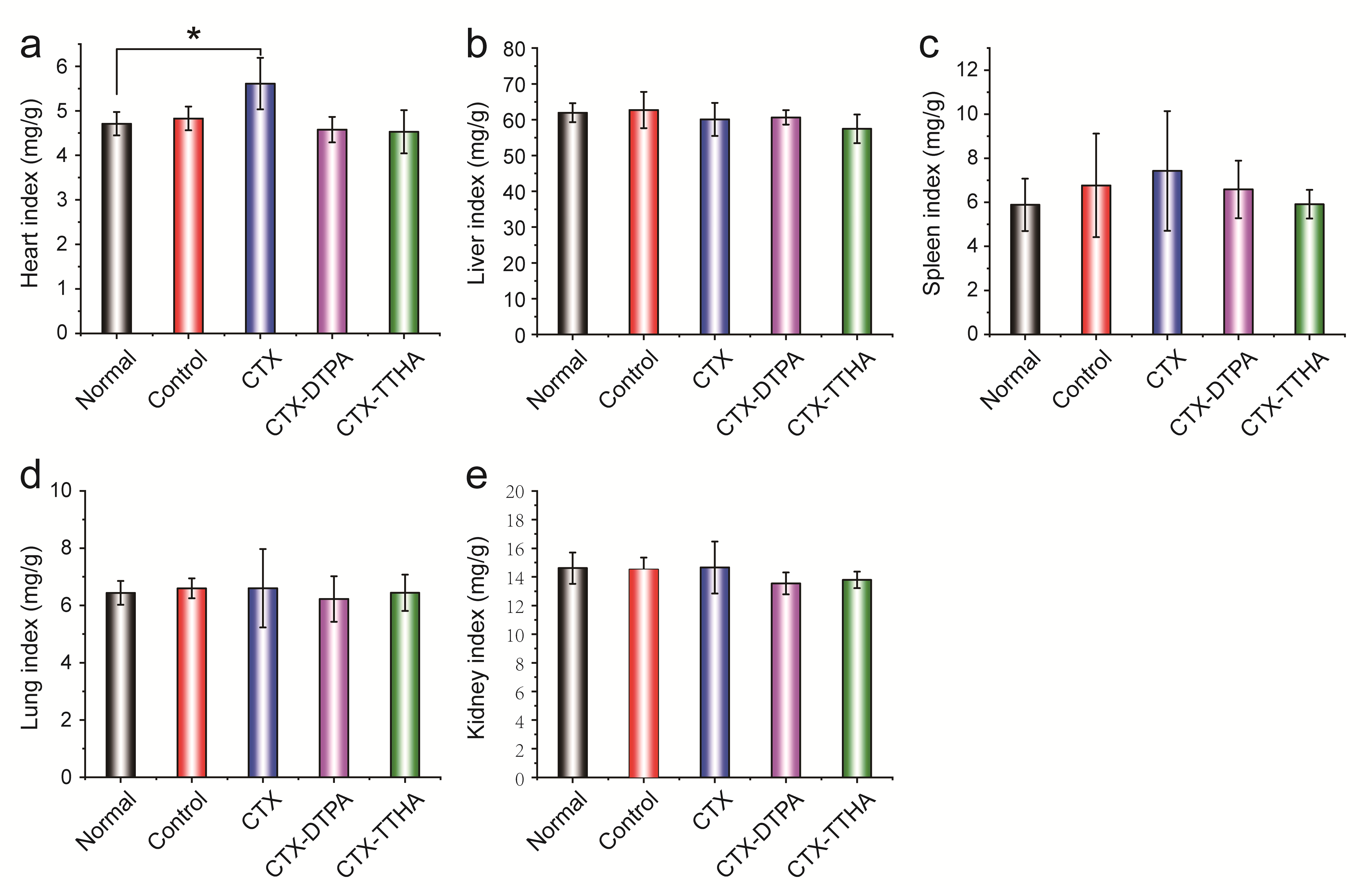


**Figure S4.** Main organ indices for heart (a), liver (b), spleen (c), lung (d), kidney (e) of mice in different groups of MCF-7 tumor bearing mice. Data were presented as mean ± SD (n=6).


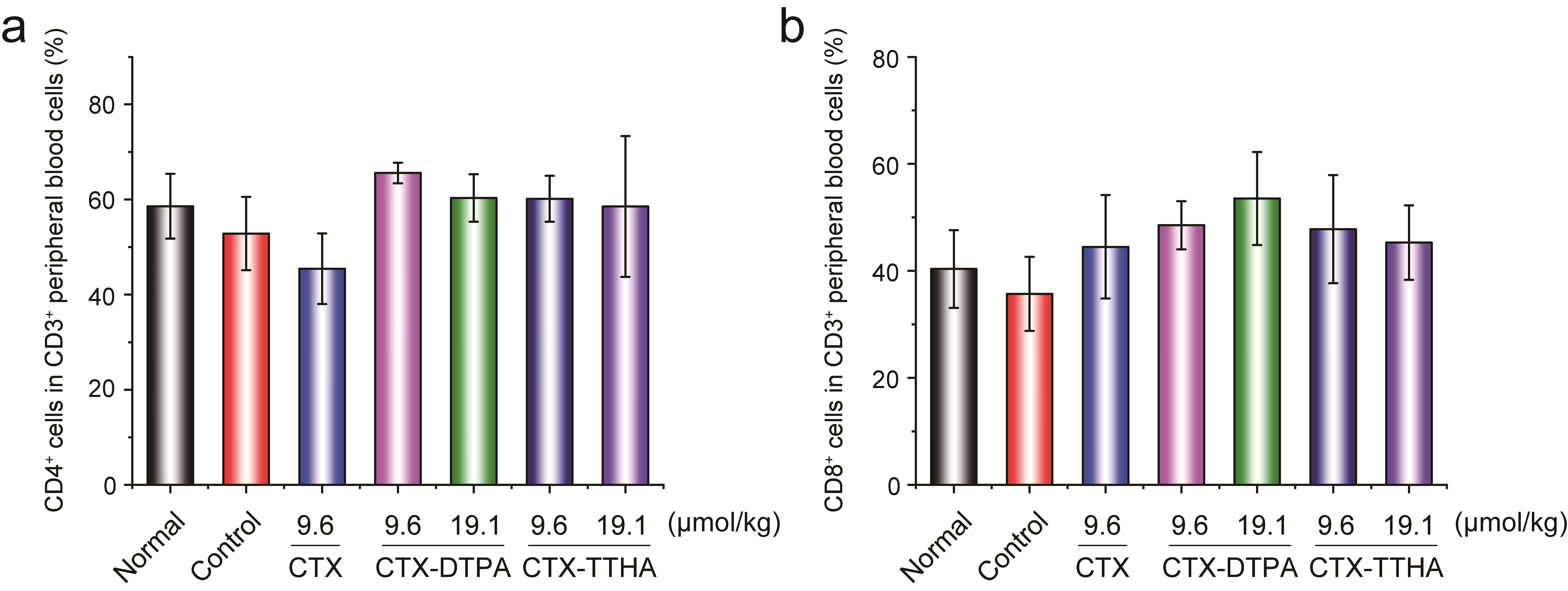


**Figure S5.** Analysis of peripheral T-cell populations in immunocompetent H22 tumor-bearing mice. (a) Proportion of CD4⁺ T cells and (b) proportion of CD8⁺ T cells within the CD3⁺ T-cell population in peripheral blood. Data were presented as mean ± SD (n=6). No statistically significant differences were observed between any treatment group and the normal group (one-way ANOVA).
